# A novel interpretable and real-time dengue prediction framework using clinical blood parameters with genetic and GAN-based optimization

**DOI:** 10.3389/frai.2025.1626699

**Published:** 2025-10-30

**Authors:** Md. Ehsanul Haque, Md. Nurul Absur, Fahmid Al Farid, Jia Uddin, Hezerul Abdul Karim

**Affiliations:** ^1^Department of Computer Science and Engineering, East West University, Dhaka, Bangladesh; ^2^Department of Computer Science, City University of New York, New York, NY, United States; ^3^Centre for Image and Vision Computing (CIVC), Faculty of Artificial Intelligence and Engineering (FAIE), Multimedia University, Cyberjaya, Malaysia; ^4^AI and Big Data Department, Woosong University, Daejeon, South Korea

**Keywords:** dengue prediction, hematological features, explainable AI, genetic algorithm, data imbalance, decision trees, real-time inference, clinical decision support

## Abstract

Dengue remains a significant and critical global health concern, especially in resource-constrained and remote regions, where traditional IgG/IgM-based testing is often delayed or not conducted properly. Furthermore, conventional machine learning often exhibits minimal interpretability and misclassification, leading to major unreliability in real-time clinical decisions. To tackle these hindrances, we proposed an interpretable, efficient, and novel machine learning framework that operates near real-time. It combines feature optimization using Genetic Algorithms (GA) and Generative Adversarial Networks (GAN) to address data imbalance, and enhances ubiquitous decision interpretability with Explainable AI (XAI). GA establishes the most predictive hematological features, which improve accuracy and transparency, whereas GAN-based data generation handles class imbalance, leading to enhanced generalization. On top of that, the optimized Decision Tree model attains 99.49% accuracy with a negligible computational cost of training and testing time 0.0025 s, and 0.0013 s respectively, superseding the current state-of-the-art. A web-based application implemented based on the proposed model enables real-time risk prediction with a latency of under 0.6 s. A comprehensive XAI evaluation using LIME, SHAP, Morris sensitivity analysis, permutation combination, and RFE consistently identifies WBC and platelet counts as key predictors. In numbers, XAI techniques represent that low White Blood Cell (WBC) count (< 3,700 cells/μL), platelet count (< 136,000 cells/μL), and Platelet Distribution Width (PDW < 23) are key indicators of dengue. Our proposed integrated GA-GAN-XAI framework bridges accuracy, interpretability, and real-time decision-making capability. This approach is highly accurate, robust for healthcare, and a highly deployable solution for dengue risk prediction for clinical dengue risk assessment.

## 1 Introduction

Dengue fever is an on-the-rise public health issue in tropical and subtropical regions such as Bangladesh, where the number of cases and deaths due to the disease is on the rise. Bangladesh recorded 321,179 dengue cases and 1,705 deaths in 2023, the deadliest outbreak in the country (Hasan et al., 2025). The most significant number of cases and fatalities happened in Dhaka city. Between consecutive years from 2000 to 2022, the country's total cases were 244,246, while the total deaths were 849 cases (Khan et al., 2024). In early 2024, Bangladesh registered more than 93,000 dengue cases and above 500 deaths; Dhaka remains the primary contributor to the fatalities (Alam, 2024). In the first 9 months of 2024, there have been 32,082 cases and 166 deaths, together with a confirmed case fatality rate of around 0.53%, in line with the high case fatality rate of 2023 (Allport, 2024). As of April 2024, there have been over 7.6 million cases of dengue and more than 3,000 deaths across the world, with the most significant number of cases reported from the Americas region. The global CFR stands at approximately 0.05% (World Health Organization, 2024a,b).

Given the concerning statistics, there is an urgent need for a more effective and efficient framework for dengue prediction and risk management, as the frequency and severity of outbreaks have escalated. Accurate predictive models are essential for enhancing preparedness and response to future epidemics. Recent advancements in rapid diagnostic tests, such as the NS1 test and the detection of IgM and IgG antibodies, have significantly improved the identification of dengue-infected patients, allowing for earlier intervention (Hasan et al., 2024; Casenghi et al., 2018). However, predicting the risk of severe dengue or its outcomes remains challenging, especially in low-resource settings, highlighting the need for better risk assessment methods. Existing dengue risk prediction models that use machine learning techniques often suffer from high misclassification rates, lack interpretability, and do not support real-time decision-making. These models typically rely on direct diagnostic tests and demographic data, overlooking more comprehensive factors, such as clinical blood parameters, that could improve prediction accuracy. Furthermore, the inability of these models to elucidate their predictions to clinicians, whether positive or negative, presents a significant barrier to their practical clinical application (Nova et al., 2021).

This paper introduces a robust and interpretable risk prediction framework based on clinical blood parameters in a decision tree-based architecture. The proposed architecture integrates Genetic Algorithms (GA) for feature extraction, Generative Adversarial Networks (GAN) for handling class imbalance, and model optimization to enhance predictive performance. Explainable AI (XAI) techniques promote transparency and dependability in healthcare applications. XAI facilitates a deeper understanding of the models used, enabling practitioners to trust the conclusions drawn from AI-based insights. Moreover, for enhanced usability and accessibility, the final model is provided as a web application. The platform is designed to be intuitive and user-friendly for different healthcare professionals, facilitating better adoption and integration into clinical practices.

This study makes several key contributions to the field of dengue risk prediction:

- Introduces an interpretable novel decision tree-based model for dengue risk prediction, incorporating clinical blood parameters such as platelet count, hemoglobin, and white blood cell count to improve the precision and robustness of the model.
- Applies Genetic Algorithms (GA) for feature selection, which optimizes the model by reducing time complexity, enhancing efficiency, and improving prediction quality by selecting the most relevant features.
- Utilizes Generative Adversarial Networks (GANs) to address the class imbalance problem by generating synthetic data, ensuring that the model is trained on a balanced dataset, which improves its generalization capabilities.
- Implements tuned models to reduce misclassification rates, optimize model performance, and ensure better predictive accuracy.
- Explainable AI (XAI) methods, such as SHAP, LIME, etc., are used to make the model's predictions interpretable to ensure healthcare professionals can trust and understand the reasoning behind the predictions.
- Morris's sensitivity analysis and the importance of permutation are performed to evaluate the contribution of each feature to the predictive performance of the model.
- SHAP and RFE are used to discuss the transferability and ubiquity of selected hematological characteristics—WBC count, platelet count, and PDW—to show that they are consistently highly predictive and could be applied to other datasets as well.
- Develop a web-based application for real-time automated dengue risk assessment, making the prediction model accessible and usable in clinical and field settings.

This study contributes to a more efficient and accurate framework for predicting dengue risk, enabling rapid and reliable decision-making for resources. Through clinical parameters, optimized features, and model interpretability, this research attempts to significantly improve dengue outbreak management and prevention.

In the remaining part of the paper, we review related work in this area in Section 2. The method for this experiment is introduced in Section 3. The results and discussion are provided in Section 4, and the conclusions and future directions are provided in Section 5.

## 2 Literature review

This section reviews the limitations of machine learning approaches and diagnostic techniques used for detecting dengue based on the existing literature and the limitations of the studies. Table 1 summarizes the key aspects of previous works, including their achieved accuracy, noted limitations, and the presence of XAI integration or web-based deployment.

**Table 1:** Summary of existing machine learning studies on dengue detection.

**Study**	**Year**	**Best model**	**Accuracy**	**Data type**	**Limitation**	**XAI**	**Real-time**
Abdualgalil et al. (2022)	2022	Extra tree	99.12%	Tabular	Dataset lacks size/diversity info; no explainability; uses direct test parameters (IgG/IgM).	No	No
Mayrose et al. (2023)	2023	SVM + MobileNetV2	95.74%	Image	Small dataset, limited generalization, no XAI, no real-time support.	No	No
Bohm et al. (2024)	2024	DT, MLP	98%	Tabular	Limited outlier handling, no XAI, unclear features used, no real-time capability.	No	No
Ming et al. (2022)	2022	XGBoost	AUC 0.86	Tabular	Missing data prep details, no explainability methods used.	No	No
Madewell et al. (2025)	2025	CatBoost	AUC 97.1%	Tabular	Incomplete feature description, unclear balancing, no real-time tool.	No	No
Riya et al. (2024)	2023	Stacking ensemble	96.88%	Tabular	Small dataset, high cost, lacks feature interaction, no real-time deployment.	Yes	No
Al-Hagree et al. (2023)	2022	Decision tree	93.7%	Tabular	Small dataset, missing preprocessing/balancing steps, no XAI.	No	Yes

The study by Abdualgalil et al. (2022) addresses the need to develop timely dengue diagnosis in areas with endemic regions, such as Yemen. The prediction accuracy was therefore improved by the authors using several machine learning algorithms, as well as the *ExtraTree method* for feature selection. The Extra Tree Classifier results in a high accuracy of 99.12% among models. The limitation of the study is not entirely explicit, as the dataset in use does not mention its size or diversity. Moreover, the paper does not incorporate explainable AI to explain how the model makes its decisions. The chosen features may not be suitable for predicting dengue, as IgM and IgG are the direct test parameters. Mayrose et al. (2023) propose a machine learning approach for detecting dengue based on peripheral blood smear images, focusing on the characteristics of platelets and lymphocytes. *A blob detection algorithm-based system* is used to find thrombocytopenia; on top of that, various classifiers are used, with the majority of classifiers for thrombocytopenia being SVM and DT, with 93.62% accuracy. In combination with SVM, MobileNetV2 deep learning features provide an accuracy of 95.74%. However, this study lacks generalization because the small dataset lacks independent validation, and its applicability to different clinical settings and diverse populations is limited by the study's focus on specific cell types and image quality. Moreover, the study does not include Explainable AI (XAI) to increase the model's interpretability by making it more trustworthy, as well as lack of real time detection method.

Bohm et al. (2024) conducted a study using machine learning techniques to improve dengue case screening in Brazil, a significant public health issue. Clinical variables from Brazil's National Notifiable Diseases Surveillance System was analyzed for data. *Mutual information* was used for feature selection, and several machine learning models were trained. Among all models, decision trees and MLP achieved an accuracy of 98%. However, there are some limitations to dealing with outliers, including a lack of explainable AI (XAI) for model transparency, real-time detection capabilities, and a lack of information about the specific features used in the models.

In this study, Ming et al. (2022) developed a supervised machine learning model to enhance the diagnosis of dengue in patients with acute febrile illnesses. The limitation of traditional point of care tests are that they do not yield optimum results. This was performed using a *gradient boosting model (XGBoost)* on prospectively collected data from Vietnam, using clinical features to predict dengue diagnosis. The dynamic threshold approach was applied to the model, which achieved an AUC-ROC of 0.86 and had a negative predictive value of >90%. Yet, this study had several limitations, including no data preparation details, a limited dataset, and a lack of explainable AI methods that could improve model interpretability.

To evaluate machine learning (ML) models for predicting severe dengue disease in Puerto Rico, Madewell et al. (2025) used Sentinel Enhanced Dengue Surveillance System data. The best performance was given by *boosting models (CatBoost, XGBoost, LightGBM), and CatBoost* achieved an AUC-ROC of 97.1%. Hemoconcentration, days post-symptom onset, and leukopenia were all key predictors. Traditional warning signs were outperformed by ML models that showed very high sensitivity and specificity. There was no detailed description of the features, the preprocessing steps were not extensively explained, and the class balancing techniques were unclear. In addition, no real-time detection system was included, which limited the clinical applicability.

Riya et al. (2024) developed an AI-based dengue detection system based on CBC data of 320 patients hospitalized in Dhaka, Bangladesh, during the 2023 outbreak. The authors claimed a good stellar result, stating that a stacking *ensemble classifier* outperformed other models with 96.88% accuracy and an F1 score of 0.9646. The key predictors were selected using feature selection and LIME-based explainability. Nevertheless, there are also some limitations, such as a small dataset, the high computational cost of the stacking model, SelectKBest for feature selection not considering feature interactions, and the lack of a real-time detection tool.

A machine learning approach to diagnosing dengue fever through an Android application is presented by Al-Hagree et al. (2023). They stated that the decision tree algorithm produced the maximum accuracy of 93.7% compared to others like the Naïve Bayes and K-NN methods. The evidence has been gathered from a minimal dataset of only 102 individuals, which may limit the generalizability of the findings. Furthermore, the selected features may not describe the full complexity of dengue disease symptoms, and there exist no explainable AI techniques to explain why the model makes certain decisions. This also lacks adequate data preprocessing and data balancing strategies to prevent the model from performing poorly overall.

In terms of related studies in this domain over the years, there are fundamental research gaps such as a lack of generalizations (Barbiero et al., 2020), limitations in data (Absur, 2022), a lack of real-life use cases (Bhardwaj et al., 2025), and a lack of explainability (Kaba Gurmessa and Jimma, 2024). Our proposed method will address all the shortcomings to make a robust, accurate, and deployable model.

## 3 Methodology

In this section, a detailed and constructive approach is outlined to efficiently predict Dengue disease.

### 3.1 Data collection

This study used a dataset from Mendeley Data (Mim et al., 2024; Assaduzzaman et al., 2024). It contains clinical blood parameters for Dengue fever. It had eight features in total. There were two demographic features, six clinical parameters, and one target class. Predicting and diagnosing Dengue fever is dependent on these features. The dataset includes 1,003 records (669 dengue-positive, 320 non-dengue) from patients at Upazila Health Complex, Kalai, Jaipurhat, Bangladesh. Clinical verification was ensured through healthcare professional involvement (Assaduzzaman et al., 2024). Order dependency has been addressed to eradicate bias from the data (Ahmed Nasif et al., 2019). The Table 2 describes each feature in the dataset.

**Table 2:** Feature descriptions of the dataset.

**Feature**	**Description**
Age	Patient's age in years.
Sex	Gender of the patient (Male/Female).
Hemoglobin	Hemoglobin levels (g/dL).
WBC count	Total white blood cell count (10^3^/μ L).
Differential Count	Distribution of WBC types (e.g., neutrophils, lymphocytes).
RBC panel	Red blood cell count and morphology analysis.
Platelet count	Total platelet count (per μ L).
PDW	Platelet distribution width (size variability).
Label (Target)	0: Not Dengue, 1: Dengue.

### 3.2 Preprocessing

#### 3.2.1 Categorical encoding

The dataset includes one categorical feature: *Sex*. This feature has been encoded using Label Encoding, where the value for Females is assigned as zero, and the Value for Males is assigned as 1. This encoding process allows us to effectively use the feature in machine learning models while preserving its original meaning and clarity.

#### 3.2.2 Missing value handling

**Figure 1:**
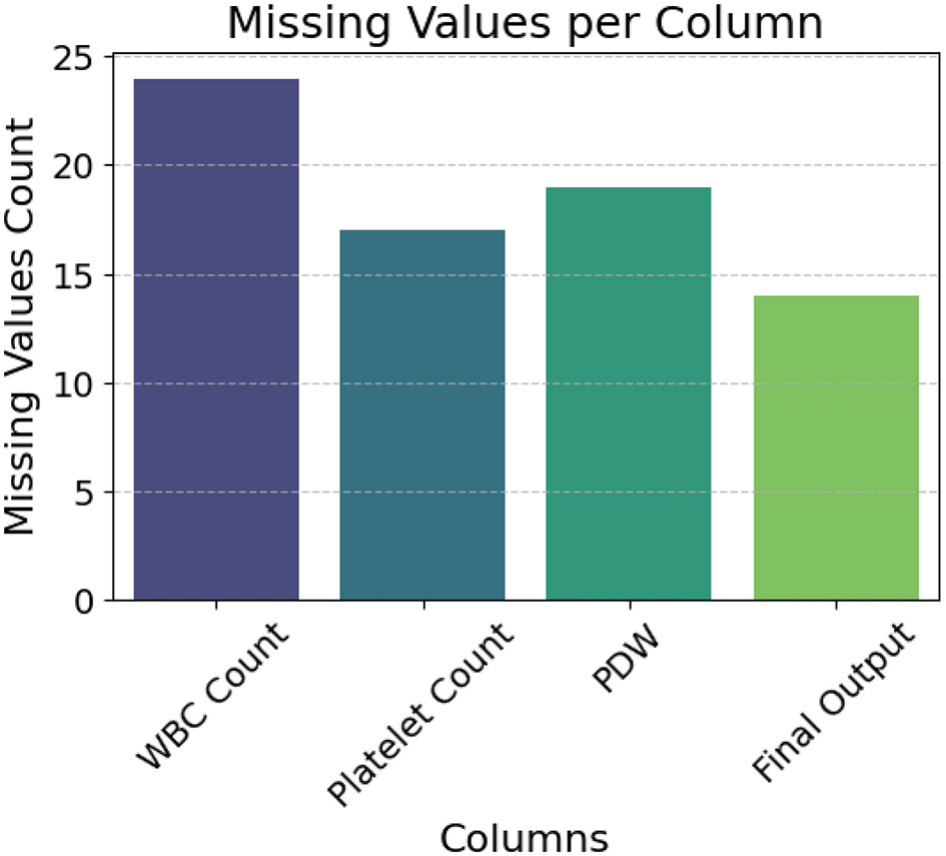
Column-wise distribution of missing values.

Figure 1 highlights missing entries in the *WBC Count, Platelet Count, PDW*, and *Final Output* columns. To address this, we applied a standard *k*-Nearest Neighbors (k-NN) imputation method for the clinical features. This approach was selected to preserve inter-feature relationships using local similarity in the patient data, and was implemented with default settings commonly used in biomedical preprocessing pipelines. Given our emphasis on building an end-to-end real-time system rather than fine-tuning individual preprocessing components, we prioritized stability and reproducibility over hyperparameter optimization.

For the target column, *Final Output*, we followed established supervised learning guidelines by removing rows with missing labels to avoid introducing bias during training or evaluation.

#### 3.2.3 Outlier handling

IQR is calculated to identify which features have the most extreme values, as shown in Table 3 (Dash et al., 2023). It reveals that Age, WBC Count, PDW, and Platelet Count have the most extreme values. Consequently, outliers are identified using the interquartile range (IQR), and adjustments are made through Winsorization capping to mitigate the impact of these outliers on the model (Blaine, 2018). Algorithm 1 provides the outlier detection and removal process.

**Table 3:** Interquartile range (IQR) analysis and feature spread comments.

**Feature**	**IQR**	**Comment**
Age	28.00	High variability; possible outliers.
Sex	1.00	Minimal spread; no notable extremes.
Hemoglobin	2.40	Moderate variability.
WBC count	3,200.00	High spread; likely outliers.
Differential count	0.00	Uniform values; very low variation.
RBC panel	0.00	Stable; no apparent spread.
Platelet count	1,16,500.00	High variability; potential extreme values.
PDW	14.20	Wide distribution; high spread.
Target (Label)	1.00	Binary class output.

**Algorithm 1:**
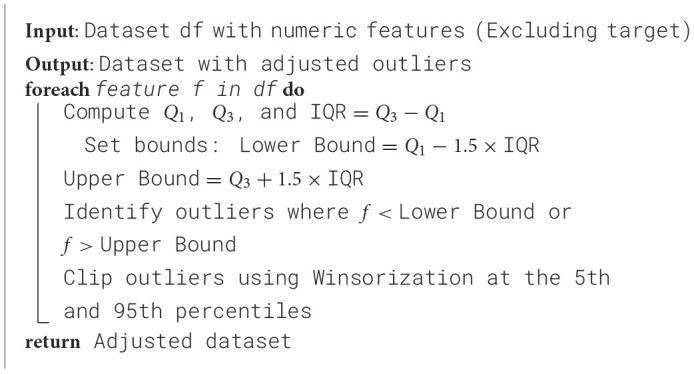
Outlier detection and adjustment using IQR and winsorization.

#### 3.2.4 Feature significance

Mutual information scores and one-way ANOVA were computed to identify the most significant features for predicting Dengue fever (Kim, 2017). A 95% confidence level ANOVA test was conducted at a 0.05 significance value threshold for p values.Sex and Hemoglobin were found to have mutual information scores of 0.0 and 0.017, respectively, with corresponding p-values of 0.3653 and 0.8368. These results indicate that Sex and Hemoglobin do not contribute significantly to the prediction of Dengue fever. The Table 4 shows mutual information scores and p-values for all features, along with their significance status.

**Table 4:** Feature selection analysis: mutual information scores and ANOVA results.

**Feature**	**MI score**	***p*-value**	**Significance**
WBC count	0.61	0.000000	Significant
Platelet count	0.59	4.73 × 10^−235^	Significant
PDW	0.31	6.13 × 10^−50^	Significant
RBC panel	0.07	1.98 × 10^−8^	Significant
Differential count	0.04	2.64 × 10^−8^	Significant
Age	0.03	2.78 × 10^−6^	Significant
Sex	0.00	0.3653	Not Significant
Hemoglobin	0.02	0.8368	Not Significant

#### 3.2.5 Data scaling

The large gap between the minimum and maximum values, and the high standard deviation value recorded in Table 5, were addressed through dataset standardization. This ensures that all the features have a mean of 0 and a standard deviation of 1 (GeeksforGeeks, 2023). SVM and KNN are sensitive to the scale of the data, and thus, standard information is essential. Once the features are scaled, the model performance improves with less training time.

**Table 5:** Feature statistics (min, max, and standard deviation).

**Feature**	**Min**	**Max**	**Std dev**
Age	40.0	120.0	20.94
Sex	2.0	2.0	0.56
Hemoglobin	11.0	25.0	1.48
WBC count	2,000.0	10,900.0	2,322.65
Differential count	0.0	1.0	0.24
RBC PANEL	0.0	1.0	0.24
Platelet count	10,000.0	5,00,000.0	88,991.46
PDW	1.0	215.0	14.58

### 3.3 Feature selection

Feature selection was performed to improve efficiency and reduce computational time. This is done with the help of a Genetic Algorithm (GA) because GA can effectively choose and minimize overfitting on a set of relevant features (Siedlecki and Sklansky, 1989). GA's ability to explore various feature subsets makes it possible to select the most meaningful features for the model. Unlike traditional methods, GA can generate a better solution to feature subsets by converging to an optimal feature subset over generations (Absur et al., 2024).

**Figure 2:**
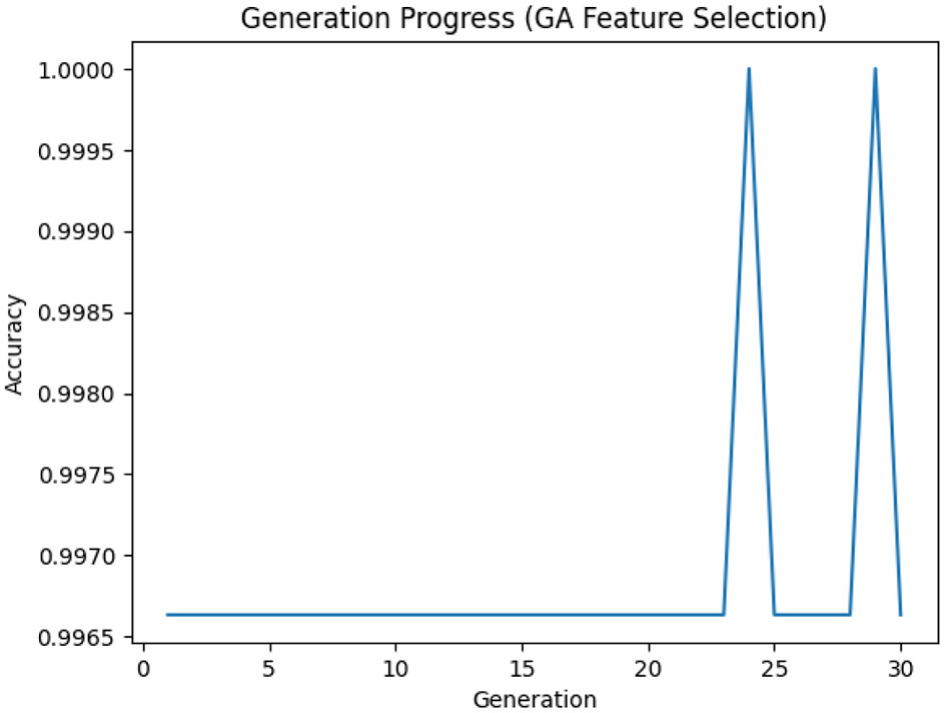
Generation progress across iterations in the genetic algorithm.

Each setting used for the GA-based feature selection is listed in Table 6. Also, Figure 2 shows the evolution of improvement in the fitness score value across generations, indicating the algorithm's convergence. The whole feature selection process is shown in Algorithm 2. The most relevant features picked out (after executing the GA) for predicting Dengue fever were WBC Count, Platelet Count, and PDW.

**Table 6:** Settings for genetic algorithm feature selection.

**Parameter**	**Value**
Number of generations	30
Population size	40
Crossover probability	0.6
Mutation probability	0.05
Tournament size	3

**Algorithm 2:**
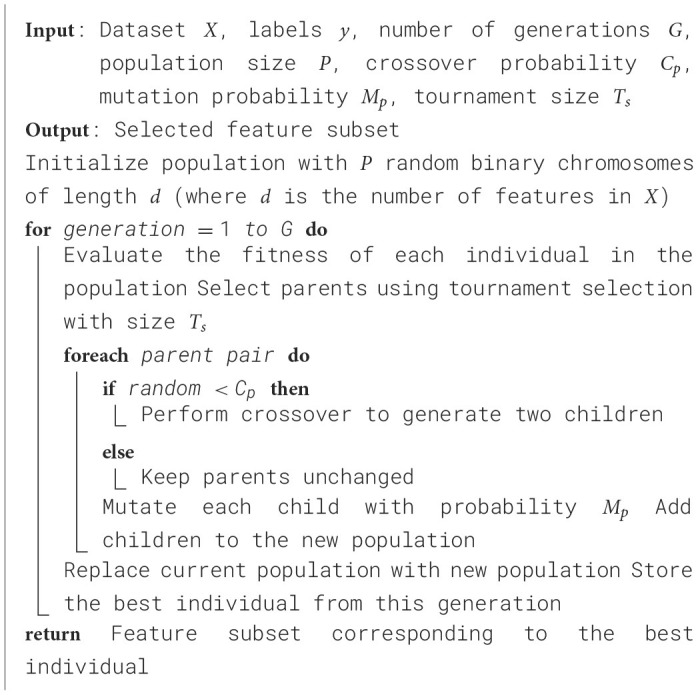
Genetic algorithm for feature selection.

### 3.4 Data splitting and cross-validation strategy

In order to have a robust evaluation of the model, an 80-20 train-test split is implemented; 80% of the data is used to train the model and 20% as the test data. This approach helps to have a good amount of training data and a separate set of test data for evaluation and checking the model's performance and ability to generalize to data it has not seen before. Stratified k-fold cross-validation was also used during training to have a robust and generalizable model evaluation. The approach retains the class distribution of each fold and avoids the bias that might arise due to non-equal data division, and it reduces the drop to an observed model estimation. After data split, the training and testing datasets are shown in Table 7.

**Table 7:** Final output distribution in training and test sets.

**Dataset**	**Final output = 1**	**Final output = 0**
Training set	535	256
Test set	134	64

#### 3.4.1 Handling class imbalance

Table 7 shows that the training set is highly imbalanced, and hence, the model predictions will be biased, and the generalization performance will degrade. To resolve this issue, a Generative Adversarial Network (GAN) is used to generate realistic synthetic samples of the minority class, resulting in 535 samples for each class in the distribution (Mariani et al., 2018). In comparison, methods like SMOTE or ADASYN might still be unable to represent the complex data patterns; hence, GAN preserves the underlying structure in the data.

The parameters within the GAN were selected to introduce stability and efficiency of training. A 100 dimension of inputs balances the complexity and the diversity of generated samples. The continuation of a network includes two hidden layers of 128-neuron ReLU activation layers that are not overfitted. Output layers involved Sigmoid activations and boundaries feature values to facilitate binary classification. Learning can be done through Adam optimizer and binary cross-entropy loss. An empirical performance yielded a batch size of 32 and 1,000 training epochs, in order to converge without overfitting. The Algorithm 3 describes the balancing process, and the GAN settings are shown in Table 8.

**Algorithm 3:**
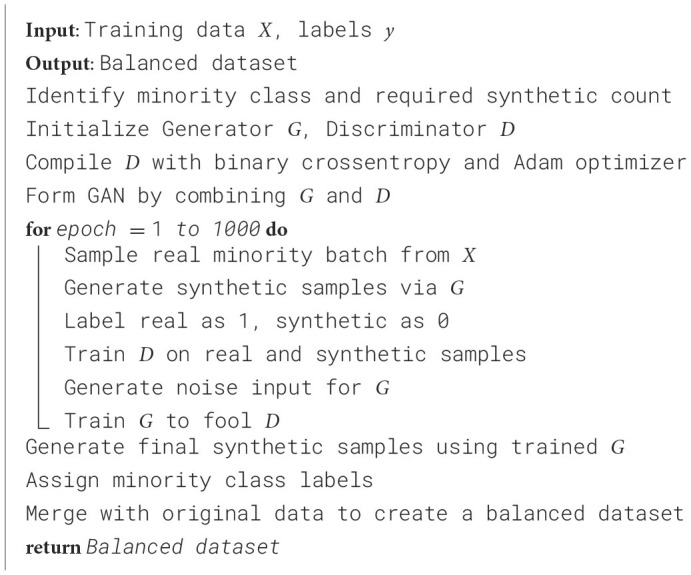
GAN-based data balancing process.

**Table 8:** GAN configuration settings.

**Parameter**	**Value**
Generator input dimension	100
Generator hidden layer	128 (ReLU)
Generator output layer	Sigmoid (feature dim.)
Discriminator hidden layer	128 (ReLU)
Discriminator output layer	Sigmoid (binary class.)
Optimizer	Adam
Loss function	Binary crossentropy
Batch size	32
Number of epochs	1,000

### 3.5 Model training

For this study, four different machine learning models with varying working principles were used, including the Support Vector Classifier (SVC), K-Nearest Neighbors (KNN), Decision Tree (DT), and Artificial Neural Network (ANN), to build a robust predictive framework for diagnosing Dengue fever. These models were selected due to their theoretical foundation, interpretability, and suitability to clinical data. This analysis highlights the predictive performance, generalizability, and reliability of each model in disease classification. Despite this, a brief overview of their working principles and selection rationale will be given in the following.

Support Vector Classifier (SVC) is a supervised learning classifier that represents the optimal hyperplane to separate classes. It can be applied to linear and non-linear data distributions or classifications (Hearst et al., 1998; Zhang, 2012). KNN is also another method that classifies new data based on the majority label of their closest neighbors, a simple yet sound approach for clinical data (Guo et al., 2003; Taunk et al., 2019). On top of that, Decision Tree makes decisions through recursively splitting the data based on feature value such as GINI or entropy, offering high interpretability and flexibility in dealing with different data types (Mienye and Jere, 2024; Lee et al., 2022). Decision Tree also reduce the risk of overfitting (Bramer, 2016). Finally, Artificial Neural Networks (ANNs), inspired by the anatomy of the human brain, can learn complex and non-linear relationships that are extremely useful in modeling intricate patterns, such as disease diagnosis (Hopfield, 1988; Jain et al., 1996). Such models have been chosen based on their theoretical background, interpretability, and the fact that they work well with clinical data. Random Forest and XGBoost are also more complex models with demonstrated powerful performance, but were not included (due to long training time and high computing capacity needs) and not currently real-time applicable in the clinical setting.

#### 3.5.1 Model interpretability

LIME is then applied to the best-performing model to explain individual predictions. Besides LIME, Morris Sensitivity Analysis and Permutation Importance are also used to see how much each feature matters. Feature weights in Morris's sensitivity analysis determine the main contributors to changes in model output (Fah et al., 2025). The study reveals essential features of their associated relationships with one another. In contrast, Permutation Importance ensures feature importance ranking by computing the performance changes resulting from randomly shuffling the feature values. This method is more reliable as it shows how much the model depends on each feature. In addition, the transferability and ubiquity of the selected features are assessed using SHAP and RFE-based feature importance techniques. By this method it is checked whether the features chosen by the GA match those selected as significant using these methods, thus ensuring consistency and robustness of the model feature selection process.

### 3.6 Web app development

A web-based application was developed using the optimal model from this research to provide real-time dengue risk assessment. The interface simulates a healthcare professional's input system and offers instant prediction by accepting key medical parameters. Built using Python and Gradio, the application ensures fast deployment, ease of use, and seamless model integration. It is hosted on Hugging Face Spaces for accessible, scalable deployment. Key implementation details—including frontend framework, backend libraries, and model integration tools—are summarized in Table 9.

**Table 9:** Web application implementation overview.

**Component**	**Description**
Platform	Hugging face spaces (multi-cloud hosting)
Frontend framework	Gradio (Python-based UI)
Backend language	Python
Machine learning model	Optimal model from this research
Key libraries	Scikit-learn, NumPy, Joblib, LIME
Functionality	Real-time risk prediction for dengue
Design philosophy	Minimal front-end code, interactive UI for healthcare usage

**Figure 3:**
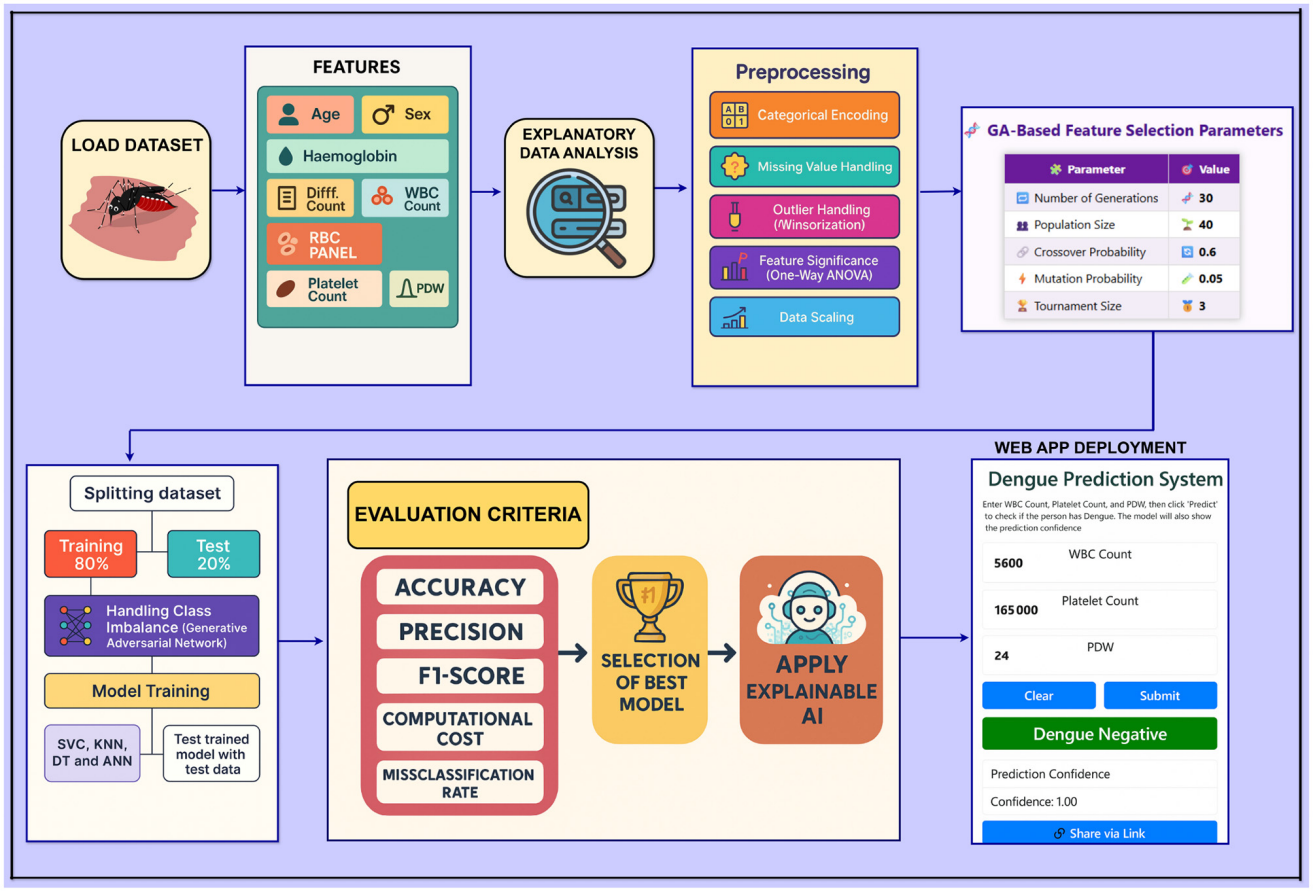
Workflow diagram of the proposed framework.

A schematic of the overall workflow of the proposed dengue prediction framework is given in Figure 3. It gives a structured and neat view of every step of the methodology, including data acquisition and processing, model training, and evaluation.

### 3.7 Evaluation metrics

The following metrics are used to evaluate model performance, providing insights into classification accuracy, reliability, and calibration through their definitions and formulas.

**Precision:** The proportion of correctly predicted positive cases out of all predicted positive cases. It is calculated as


$$\text{Precision}=\frac{TP}{TP+FP}$$

where TP is true positives and FP is false positives.

**Recall:** The proportion of correctly predicted positive cases out of all actual positive cases. It is calculated as


$$\text{Recall}=\frac{TP}{TP+FN}$$

where FN is false negatives.

**F1-Score:** The harmonic mean of Precision and Recall, providing a balance between the two. It is calculated as


$$\text{F1}=2\times \frac{\text{Precision}\times \text{Recall}}{\text{Precision}+\text{Recall}}$$

**Confusion matrix:** A summary of prediction results that shows counts of true positives (TP), false positives (FP), true negatives (TN), and false negatives (FN). It is often represented as:


$$\left[\begin{array}{cc} TP & FN\\ FP & TN \end{array}\right]$$

where rows represent actual classes and columns represent predicted classes.

**AUC (Area under the curve):** Measures a model's ability to distinguish between classes. The value ranges between 0 and 1, with higher values indicating better discrimination.

**Cohen's Kappa:** A statistic that measures agreement between actual and predicted classifications, adjusted for chance agreement. Calculated as


$$\kappa =\frac{p_{o}-p_{e}}{1-p_{e}}$$

where *p*_*o*_ is the observed agreement and *p*_*e*_ is the expected agreement by chance.

**Brier score:** The mean squared difference between predicted probabilities and the actual outcomes, measuring the accuracy of probabilistic predictions. Calculated as


$$\text{Brier}=\frac{1}{N}\overset{N}{\underset{i=1}{\sum }}{\left(f_{i}-o_{i}\right)}^{2}$$

where *f*_*i*_ is the predicted probability and *o*_*i*_ is the actual outcome.

## 4 Results and discussion

In this section, the models to be evaluated for predicting dengue disease are presented and discussed. The results of various machine learning models are analyzed in terms of classification accuracy, reliability, and generalizability in clinical settings. The results provide insights into how real-time dengue detection can be achieved using different models to achieve high accuracy and interpretability for clinical decision making.

### 4.1 Model evaluation using training and validation performance

Table 10 presents training and validation accuracies of various machine learning models used to predict dengue. The SVC model's training accuracy reached 99.44%, and its validation accuracy was 99.35%. Regarding generalization, the KNN model achieved the highest validation accuracy of 99.53%, with a training accuracy of 99.63%. The model with the highest training accuracy was the Decision Tree, with a score of 99.81%. However, its validation accuracy was 99.35%. The ANN model also had the same consistent results, as its training accuracy was 99.53% and validation accuracy was 99.44%.

**Table 10:** Model training and validation accuracies.

**Model**	**Training accuracy (%)**	**Validation accuracy (%)**
SVC	99.44	99.35
KNN	99.63	**99.53**
Decision Tree	**99.81**	99.35
ANN	99.53	99.44

Bold values used to highlight the best-performing results.

Compared to other models, the KNN model has better performance in terms of validation accuracy, indicating its better generalization ability for dengue prediction. Despite that, the training accuracy of the Decision Tree model was the highest. Still, the validation accuracy was slightly lower, thus indicating that it might not generalize as well as the KNN.

**Figure 4:**
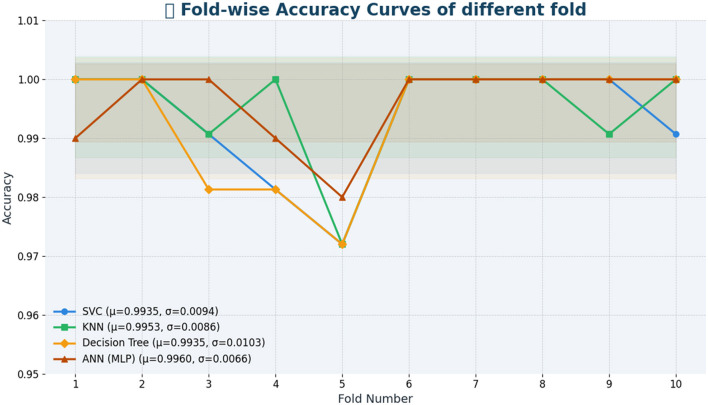
Fold-by-fold learning curves.

Table 11 shows fold-wise validation accuracies and standard deviations for each model. The SVC model yielded consistent results in each test fold, as its measurements consistently reached an accuracy of 0.99, although they fluctuated by 0.0094. In performing similarly to KNN, the model produced smaller variation outcomes (SD = 0.0086). Despite consistent performance in most splits, the Decision Tree model varies more than others, according to the 0.0103 standard deviation value. ANN demonstrates the most reliable results because its standard deviation stays at 0.0075 across all the data splits. The fold-by-fold learning curves provided in Figure 4 also support these findings with the general trends in training and validation performance across all the models indicating their stability when cross-validation was implemented.

**Table 11:** Validation accuracy (fold-wise) and standard deviation for different models.

**Model**	**Fold-wise accuracy**	**Std. Dev**.
SVC	1.00, 1.00, 0.99, 0.98, 0.97, 1.00, 1.00, 1.00, 1.00, 0.99	0.0094
KNN	1.00, 1.00, 0.99, 1.00, 0.97, 1.00, 1.00, 1.00, 0.99, 1.00	0.0086
Decision Tree	1.00, 1.00, 0.98, 0.98, 0.97, 1.00, 1.00, 1.00, 1.00, 1.00	0.0103
ANN	1.00, 1.00, 0.99, 0.98, 0.98, 0.99, 1.00, 1.00, 1.00, 1.00	**0.0075**

Bold values used to highlight the best-performing results.

Table 12 also provides precision, recall, and F1 score metrics for each model based from training dataset, aside from training and validation accuracies. The values in these metrics are crucial in understanding the model's capacity to effectively classify individual classes, significantly important in real applications like dengue prediction, where the costs of false positives and false negatives are high.

**Table 12:** Precision, recall, and F1-score for each model and class (training results).

**Model**	**Class**	**Precision**	**Recall**	**F1-score**
SVC	0	0.9944	0.9944	0.9944
	1	0.9944	0.9944	0.9944
KNN	0	**0.9981**	0.9944	0.9963
	1	0.9944	**0.9981**	0.9963
Decision Tree	0	0.9963	**1.0000**	**0.9981**
	1	**1.0000**	0.9963	**0.9981**
ANN	0	**1.0000**	0.9907	0.9953
	1	0.9907	**1.0000**	0.9953

Bold values used to highlight the best-performing results.

Finally, the SVC model optimizes performance with a precision and recall of 0.9944 for both classes, resulting in an F1-score of 0.9944. In terms of precision, the KNN model achieves a score of 0.9981 for class 0 and a recall score of 0.9981 for class 1, resulting in an F1 score of 0.9963 in both classes. Looking at the Decision Tree we can see substantial precision (1.0000) for class 1 as well as a good F1-score of 0.9981. In class 0 (1.0000) the ANN model is perfect in precision, for class 1 (0.9907) the recall is strong (0.9953) and the F1 is 0.9953.

In general, all models yield very reasonable results, especially the Decision Tree, KNN, and ANN, which demonstrate good capabilities in classifying both classes, making them suitable for predicting dengue.

### 4.2 Model evaluation using testing accuracy and AUC

The testing accuracy and AUC values of the evaluated models are depicted in Table 13. An identical testing accuracy of 98.99% was achieved by the Support Vector Classifier (SVC), K-Nearest Neighbors (KNN), and Artificial Neural Network (ANN), suggesting high precision of diagnosing between dengue and non-dengue cases. Although these three models have a uniform accuracy, there is variation in AUC. For example, the ANN achieved a perfect AUC of 1.00, indicating that it perfectly distinguishes between dengue and non-dengue cases. On the other hand, SVC and KNN produce slightly lower AUCs, about 0.9827 and 0,9883, respectively.

**Table 13:** Model testing accuracy and AUC score.

**Model**	**Testing accuracy (%)**	**AUC score**
SVC	98.99	0.9827
KNN	98.99	0.9883
Decision Tree	**99.49**	0.9964
ANN	98.99	**1.0000**

Bold values used to highlight the best-performing results.

It is also observed that among all models, the optimized Decision Tree demonstrated best performance, achieving an accuracy of 99.49% along with a near-perfect AUC of 0.9964. The model's combination of such high accuracy and strong class separability makes it a potentially very reliable clinical decision tool for dengue diagnosis.

In addition to testing accuracy and AUC, Table 14 shows the precision and F1 Score of the models. These metrics provide a deeper insight into overall performance, facilitating a more detailed evaluation of the models' effectiveness. To begin with, it is observed that the Decision Tree model stands out with perfect recall for class 0 and the highest F1 Score for class 1, highlighting its exceptional ability to identify both dengue and non-dengue cases. In contrast, the ANN demonstrates excellent precision for class 0 but exhibits a slight dip in recall. However, both SVC and KNN show stable performance, maintaining a balanced precision and recall, although their F1-scores are slightly lower than those of the Decision Tree.

**Table 14:** Precision, recall, and F1-score for each model and class (testing results).

**Model**	**Class**	**Precision**	**Recall**	**F1-score**
SVC	0	0.9844	0.9844	0.9844
	1	0.9925	0.9925	0.9925
KNN	0	0.9844	0.9844	0.9844
	1	0.9925	0.9925	0.9925
Decision Tree	0	0.9846	**1.0000**	**0.9922**
	1	**1.0000**	0.9925	**0.9963**
ANN	0	**1.0000**	0.9688	0.9841
	1	0.9853	**1.0000**	0.9926

Bold values used to highlight the best-performing results.

### 4.3 Model evaluation using confusion matrix and ROC curve

**Figure 5:**
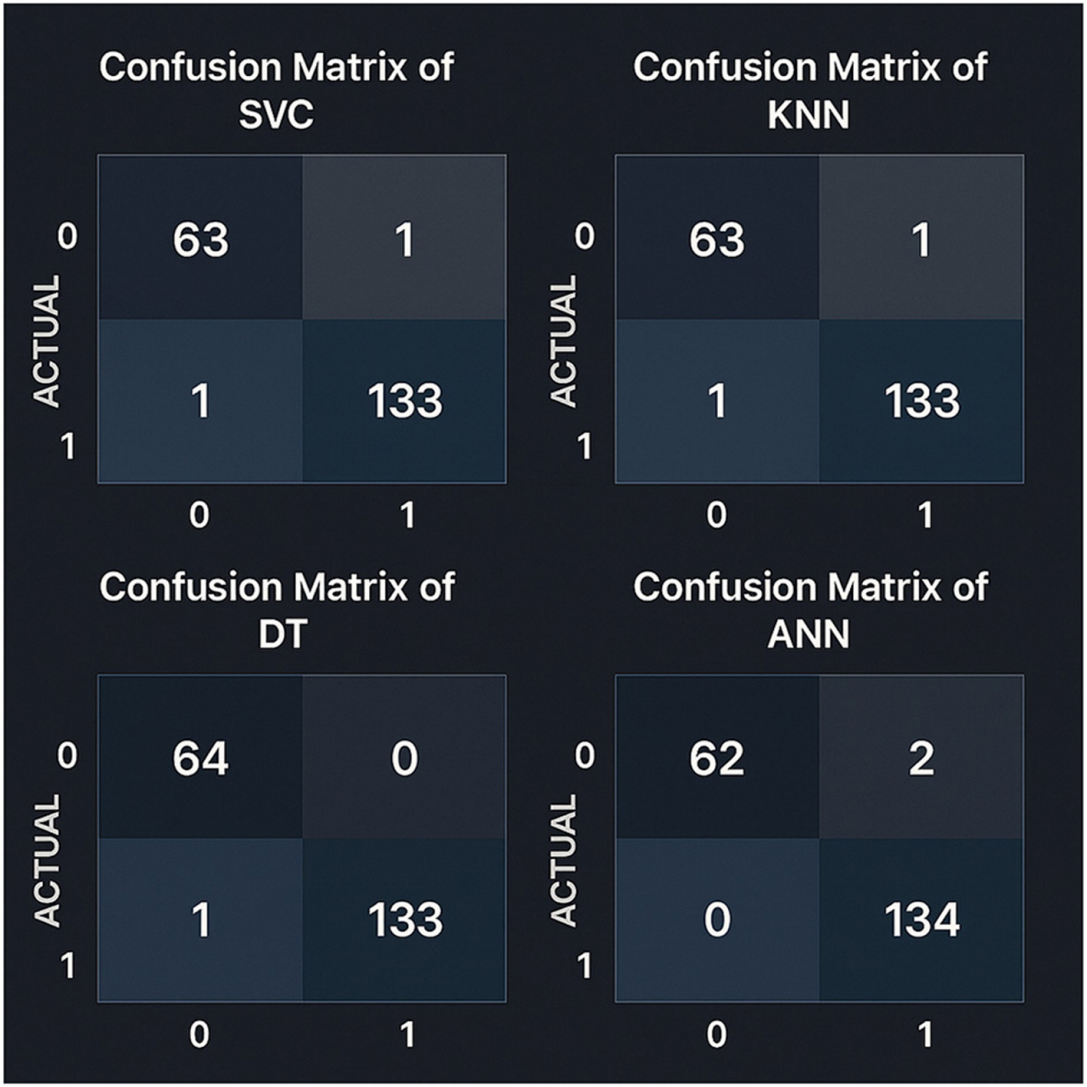
Confusion matrices for all models evaluated on the test set using an 80–20 train-test split. Precision and recall metrics were calculated with scikit-learn version 1.2.2.

To gain further insight of models behavior, we did a thorough analysis on model performance using confusion in Figure 5. Traditional metrics provide a summary of evaluation, but confusion matrices help a lot more in terms of identifying predictions, including true positives, true negatives, false positives, and false negatives. This breakdown is necessary to evaluate the extent to which a model accurately identifies positive and negative cases.

The Support Vector Classifier (SVC) and the KNearest Neighbors (KNN) models were identical, having 63 true negatives, one false positive, 133 true positives, and one false negative. In conrast, the Decision Tree model was slightly better by reducing false positives and achieving 64 true negatives, 133 true positives, and one false negative. Considering that the Artificial Neural Network (ANN) model had the best results among all the models in terms of detecting positives with 134 true positive and zero false negatives. However, it was slightly less accurate in detecting negative cases, with 62 correct and two wrong predictions. The analysis shows that the Decision Tree is a highly reliable model with only one misclassification.

This highlights the fact that models should be evaluated based on their capacity to position both classes correctly. Depending on clinical priorities, such as minimizing incorrect classifications and consistency across varied case types, the model choice is made.

**Figure 6:**
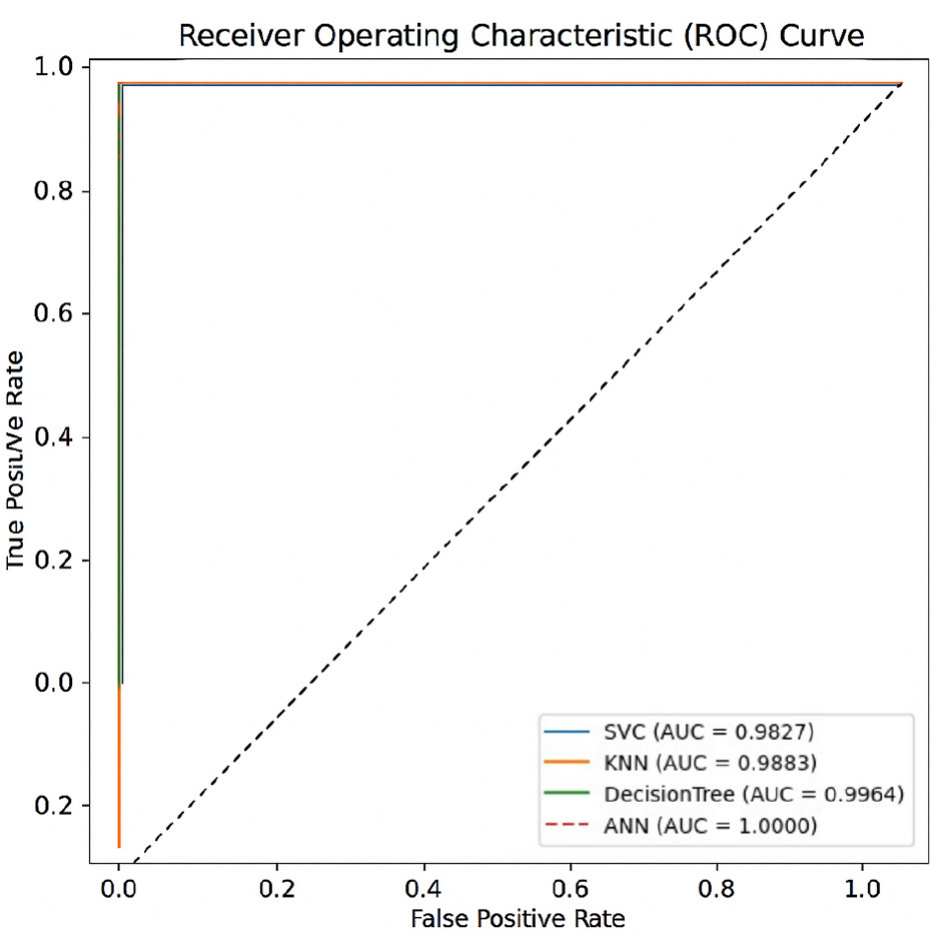
ROC curves of all models evaluated on the test set using an 80–20 train-test split. The Area Under the Curve (AUC) metrics were computed with scikit-learn version 1.2.2.

Moreover, as seen from Figure 6, the ROC curves show that the ANN model has a perfect AUC, which is more consistent with its ability to distinguish between positive and negative DENV cases. The ROC curves of the other models remain near the ideal levels, indicating excellent overall classification capabilities. These results are another validation of the robustness of the models evaluated, which would be directly applicable on the clinical level.

### 4.4 Model reliability evaluation

To ensure the reliability of the models beyond accuracy, Cohen's Kappa and Brier Score metrics are used as presented in Table 15. The accuracy of the probabilistic predictions is evaluated by the Brier Score, and a lower value indicates a better-calibrated model. At the same time, the Kappa score measures how close predicted and actual labels are to agreement, taking chance agreement into account (McHugh, 2012; Bradley et al., 2008; Viera and Garrett, 2005). Likewise, other models such as SVC, KNN, and ANN had very high Kappa values (≥0.9767), though with considerably higher Brier Score which implies a bit less confidence in prediction. These results confirm that the optimized Decision Tree model performs best in terms of reliability and consistency of prediction and can be used to make reliable and trustworthy Dengue detections.

**Table 15:** Model reliability evaluation using Kappa and Brier score.

**Model**	**Cohen's Kappa**	**Brier score**
SVC	0.9769	0.0149
KNN	0.9769	0.0125
Decision Tree	**0.9885**	**0.0050**
ANN	0.9767	0.0067

Bold values used to highlight the best-performing results.

### 4.5 Evaluation of data balancing techniques: GAN vs. SMOTE

To address the inherent class imbalance in the dataset, we evaluated two widely-used data augmentation techniques: Generative Adversarial Networks (GAN) and Synthetic Minority Over-sampling Technique (SMOTE). The objective was to assess which method produces more generalizable models in a clinical prediction setting. SMOTE generates synthetic samples by interpolating between minority class instances and their nearest neighbors. While this method is computationally efficient, it often introduces oversimplified synthetic data, which may fail to capture the true complexity of clinical feature distributions. In contrast, GAN-based augmentation learns the underlying distribution of the minority class and generates synthetic samples that better resemble real patient profiles. This generative process aims to preserve feature correlations and avoid introducing overly smooth or unrealistic instances. To ensure a fair comparison, we retrained all models using the same workflows and hyperparameters for both SMOTE- and GAN-augmented datasets. Performance metrics across training, validation, and test sets are summarized in Table 16. While SMOTE resulted in high training accuracies, the models demonstrated poor generalization to the test set, with degraded Kappa and Brier scores. This indicates potential overfitting to synthetic data. Conversely, models trained with GAN-augmented data achieved consistently high performance across all splits (as shown in Tables 10, 11, 13) and exhibited improved reliability, as reflected in higher Kappa coefficients and lower Brier scores (Table 15). These findings support the conclusion that GAN-based augmentation is more effective for balancing clinical tabular data in this context.

**Table 16:** Performance of models trained on SMOTE-balanced dataset.

**Model**	**Train Acc. (%)**	**Test Acc. (%)**	**Kappa**	**Brier score**
SVC	99.91	67.68	0.0000	0.2342
KNN	100.00	67.68	0.0000	0.3232
Decision Tree	99.16	67.68	0.0000	0.3160
ANN	99.91	67.68	0.0000	0.3232

### 4.6 Statistical tests for model comparison

To rigorously evaluate model performance, two complementary statistical tests were conducted: McNemar's test and paired t-tests.

McNemar's test was used to determine whether a significant difference exists between the predicted labels of each model and the actual ground truth. The null hypothesis (*H*_0_) assumes no difference in misclassification rate, while the alternative hypothesis (*H*_1_) suggests a significant deviation. A threshold of *p* = 0.05 was used to determine statistical significance. If the *p*-value falls below this threshold, *H*_0_ is rejected, indicating that the model's predictions differ significantly from the true labels.

Paired t-tests were applied to the cross-validation accuracy scores to assess whether differences between each model and a baseline accuracy of 1.0 are statistically significant. The null hypothesis (*H*_0_) posits no difference in mean accuracy, while the alternative hypothesis (*H*_1_) suggests a significant deviation from the baseline.

*McNemar's test was selected due to its suitability for comparing categorical outcomes in binary classification settings where predictions are paired, such as comparing model outputs against ground truth labels. The paired t-test was employed to compare repeated-measure accuracy values (from cross-validation), which provides a stable estimate of performance differences against a theoretical upper bound*.

These tests offer a robust statistical framework for evaluating model reliability and comparative effectiveness. Table 17 presents the results of McNemar's test, and Table 18 summarizes the paired t-test findings.

**Table 17:** McNemar's test results (model vs. ground truth).

**Model**	***p*-value**	**Interpretation**
SVC	0.0000	Significant difference; higher misclassification rate
KNN	0.0000	Significant difference; higher misclassification rate
Decision Tree	0.6171	No significant difference; strong agreement with ground truth
ANN	0.0000	Significant difference; higher misclassification rate

**Table 18:** Paired *t*-test *p*-values of each model vs. baseline (accuracy = 1.0).

**Model**	***p-*value**	**Significance**
SVC	0.1679	No significant difference
KNN	0.1679	No significant difference
Decision Tree	0.1108	No significant difference
ANN	0.1039	No significant difference

As shown in Table 17, the Decision Tree model demonstrates the closest alignment with the ground truth, indicating strong predictive consistency.

The paired t-test results confirm that none of the evaluated models show statistically significant deviation from a perfect accuracy baseline, indicating comparable mean performance.

While statistical tests were focused on accuracy-based metrics, other evaluation indicators—such as F1-score and AUC (Tables 12, 13)—were also taken into consideration for a holistic comparison. These metrics are especially relevant for class imbalance and threshold sensitivity, but not all lend themselves directly to paired significance testing. Therefore, they were used descriptively, not inferentially.

When combined with results from training/testing time (Table 19), these insights reinforce the selection of the Decision Tree model. Despite ANN achieving slightly higher AUC, the Decision Tree offers more reliable alignment with ground truth (as shown by McNemar's result), comparable performance in paired tests, lower inference time, and enhanced interpretability—making it the most balanced and practical choice for deployment.

**Table 19:** Model training and testing time analysis.

**Model**	**Training time (s)**	**Testing time (s)**
SVC	0.0259	0.0027
KNN	**0.0025**	0.0183
Decision Tree	0.0029	**0.0013**
ANN	20.4911	0.1141

Bold values used to highlight the best-performing results.

### 4.7 Time complexity analysis

Now, looking at the complexity, Table 19 compares the training and testing times of each model. The Support Vector Classifier (SVC) is efficient, with training and testing times of 0.0259 seconds and 0.0027 seconds, respectively. In comparison, the K-Nearest Neighbors (KNN) is the fastest in training, requiring just 0.0025 seconds. However, its testing time is comparatively higher, at 0.0183 seconds. However, the Decision Tree offers a good compromise between speed and performance as it takes 0.0029 seconds for training and 0.0013 seconds for testing. In comparison, the Artificial Neural Network (ANN) is relatively slow, requiring 20.4911 seconds for training and 0.1141 seconds for testing.

Finally, in summary, both the Decision Tree and KNN models are better in terms of computational cost. At the same time, although the ANN can reach higher accuracy, it consumes higher computational resources that make it unsuitable for real-world applications.

Table 19 shows each model's training and testing times.

### 4.8 Best model for prediction of dengue

The tuned Decision Tree model was chosen to be the most effective and efficient approach for dengue prediction, after complete evaluation of the performance of model through the following key performance metrics: training accuracy, validation accuracy, test accuracy, confusion matrix, AUC score, training time, testing time, Matthews Correlation Coefficient, Cohen's Kappa, and Brier score. It provides a high test accuracy of 99.49% with only one misclassification and therefore high reliability and low error. In addition, it has swift training and testing times, making it well-suited for real-time clinical applications. Furthermore, McNemar's test further confirms the Decision Tree as the best model, showing no significant difference from the ground truth predictions. It is found that the Decision Tree model performs exceptionally well, and its strong generalization to unseen data is credited to proper hyperparameter tuning. Furthermore, the effective use of genetic algorithm-based feature selection and data balancing, combined with Generative Adversarial Networks (GAN), has significantly improved the model's robustness and predictive performance.

**Figure 7:**
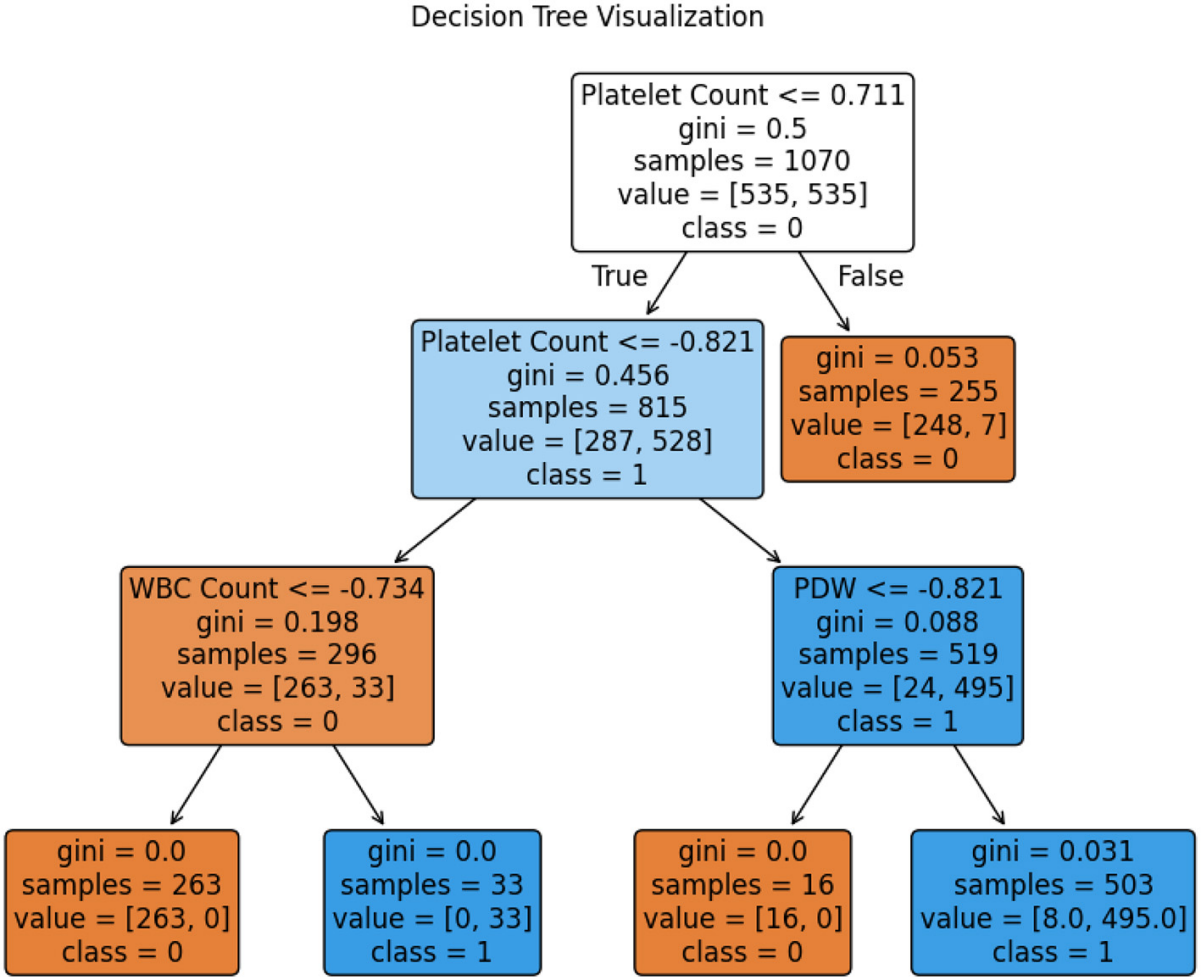
Visualization of the tree of random forest.

The comprehensive evaluation metrics and the Decision Tree visualization are presented in Table 20 and Figure 7, respectively. Also, the Decision Tree model was then configured with the hyperparameter set as shown in Table 21 and produced the trained parameters as shown in Table 22. This information ensures that anyone can be reproduced the results.

**Table 20:** Evaluation metrics of the optimized decision tree model.

**Metric**	**Value**
Training accuracy	99.81%
Validation accuracy	99.35%
Testing accuracy	99.49%
AUC score (test)	0.9964
Cohen's Kappa	98.86%
Brier score	0.0050
Training time (s)	0.0025
Testing time (s)	0.0013

**Table 21:** Decision tree classifier: hyperparameters.

**Hyperparameter**	**Value**
ccp_alpha	0.01
class_weight	None
criterion	gini
max_depth	5
max_features	sqrt
max_leaf_nodes	None
min_impurity_decrease	0.0
min_samples_leaf	5
min_samples_split	10
min_weight_fraction_leaf	0.0
monotonic_cst	None
random_state	42
splitter	best

**Table 22:** Decision tree classifier: trained parameters.

**Trained parameter**	**Value**
Max depth (trained)	4
Number of leaves	7
Number of features	3
Number of outputs	1
Number of classes	2
Classes	[0, 1]
Feature importances	[0.4219, 0.2925, 0.2856]
Tree node count	13

### 4.9 Interpreting model decisions with XAI

**Figure 8:**
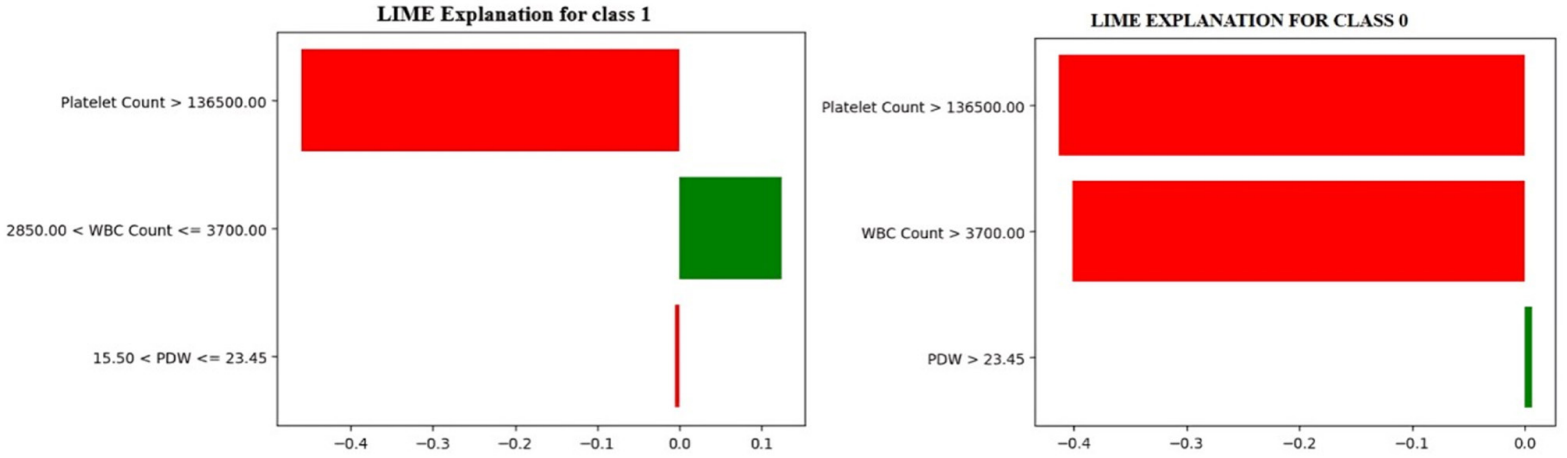
LIME local explanations for class 0 & class 1.

To understand how the Optimized Decision Tree model makes a prediction, LIME (Local Interpretable Model-agnostic Explanations) is used to obtain insight into the contribution of each feature affecting the prediction of class 0 and class 1. It approximates the model's behavior to help explain its decision-making process via a simpler, interpretable model. This study uses LIME to explain the predictions for the 0th and 11th indices of the test dataset, which are classified as class 1 (Dengue) and class 0 (No Dengue), respectively. Figure 8 shows the local LIME explanation for both Class 0 and Class 1.

In Figure 8, the model predicts a non-dengue (Class 0) case, whereas the LIME explanation points out that when the platelet count is greater than 136,000, the white blood cell (WBC) count is more than 3,700, and the platelet distribution width (PDW) exceeds 23.5, these features together contribute most significantly to the model's decision to classify the case as non-dengue. Conversely, it shows an instance classified as dengue-positive (Class 1). The rationale states that the critical variables influencing the model's prediction toward a positive dengue outcome are platelet counts less than 136,000, WBC counts between 2,850 and 3,700, and PDW values ranging from 15.50 to 23.5.

These findings relate the model to standard clinical markers of dengue infection and reinforce the need for explainable machine learning to improve transparency in the decision-making process and foster confidence in automated medical diagnosis systems (Ralapanawa et al., 2018; Chaloemwong et al., 2018; Asha et al., 2023; Shinde, 2024).

### 4.10 Sensitivity and importance analysis

Table 23 shows the results of the Morris sensitivity analysis, where Platelet Count and WBC Count have the most influence on the model's prediction. Mu* (absolute mean impact) and Sigma* (standard deviation) of the two features are high, implying that the model's output is susceptible to changes in these inputs, and some quantities that have nonlinearity or interactive effects. Moreover, changes in the values of these two features resulted in consistent drops in prediction accuracy, indicating a substantial impact on shaping model decisions. On the other hand, PDW gives zero values for both Mu* and Sigma*, it means that displaying values of the variations within this dataset did not affect the model output, taking that it also has low influence in this dataset.

**Table 23:** Morris sensitivity analysis results.

**Feature**	**μ (Mu)**	μ^*^(Mu*)	**σ (Sigma)**	σ^*^(Sigma*)
WBC count	–0.702	0.702	0.7492	0.7492
Platelet count	–0.774	0.774	0.7504	0.7504
PDW	0.000	0.000	0.0000	0.0000

μ^*^ (mu star) represents the meaning of the absolute elementary effects, indicating the overall influence of each input variable on the output. σ^*^ (sigma star) denotes the standard deviation of the elementary effects, reflecting the non-linear and interaction effects among the input variables.

Like the Morris Sensitivity analysis, the permutation importance results (Table 24) show that the most influential features are WBC Count and Platelet Count, as they have relatively high importance scores. When the values of these features are randomly shuffled, the model's performance is notably affected, reinforcing their critical role in making predictions. This is evident with the low standard deviations on both features, meaning that we tend to be consistent across different permutations in terms of their influence. On the other hand, the importance score of PDW is 0, indicating that changing its values does not affect the model's output. This further confirms the results of the Morris sensitivity analysis, which found that PDW has insignificant predictive value in this dataset.

**Table 24:** Permutation importance of top features.

**Feature**	**Importance**	**Std. dev**.
WBC count	0.2368	0.0185
Platelet count	0.2069	0.0186
PDW	0.0000	0.0000

The Morris sensitivity analysis and the permutation importance estimate how each feature, as an individual, affects the output of the model by quantifying how predictions change when the value of the feature is changed or randomly reshuffled. The two methods showed that PDW was of low importance. But Genetic Algorithm (GA) chooses the features because of their overall contribution to the model performance, and interactions that are not observed by Morris and permutation methods might not be captured. That is why GA chose PDW in spite of the fact that individually it has a low importance.

### 4.11 Exploring transferability and ubiquity of selected features

**Figure 9:**
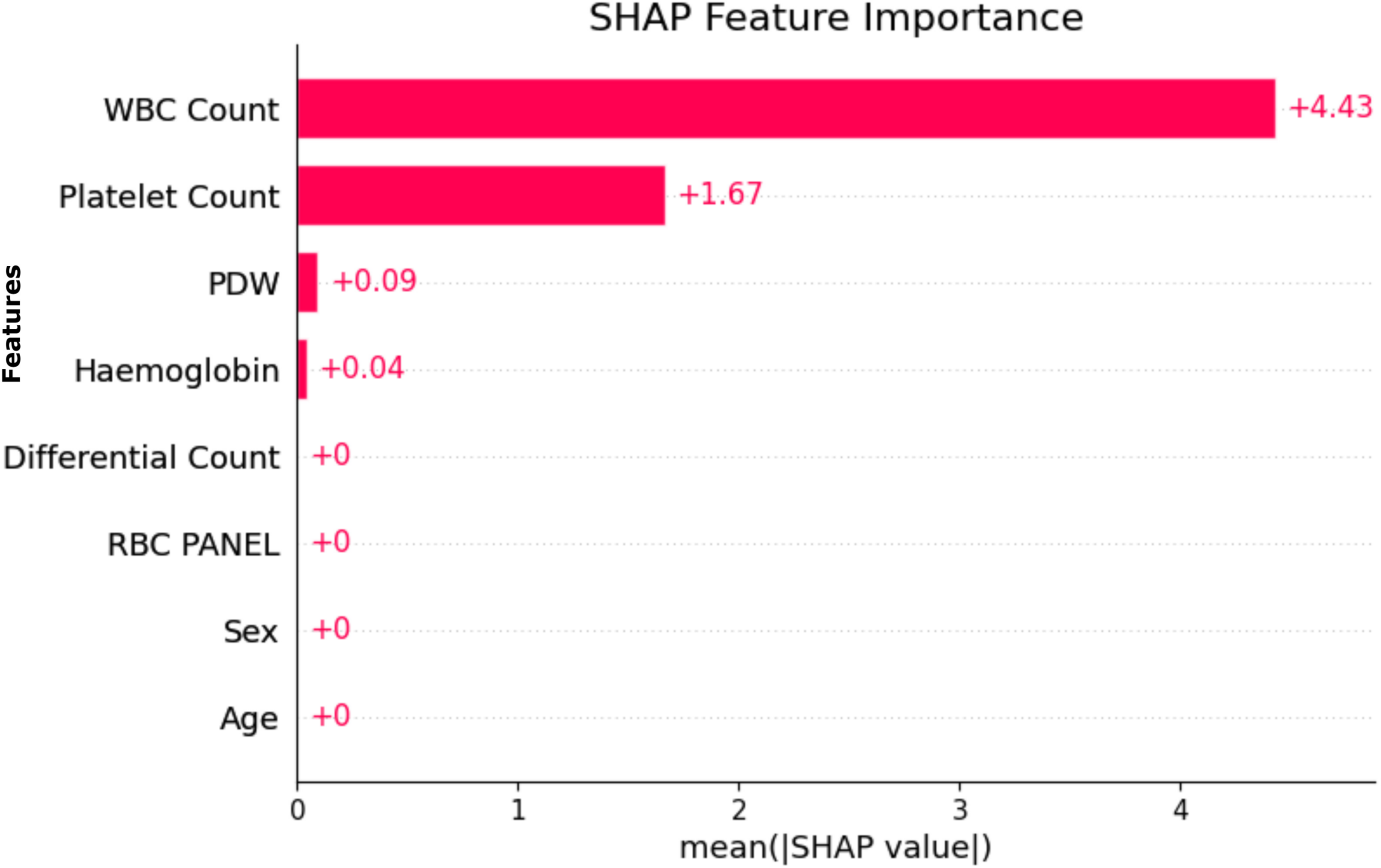
SHAP based feature importance.

As shown in Figure 9, the most important features for predicting model results, based on the SHAP (SHapley Additive exPlanations) importance plot, are WBC Count, Platelet Count, PDW, and Hemoglobin. They appeared consistently with the highest importance scores in the entire dataset, indicating their importance in distinguishing between dengue and non-dengue cases.

**Figure 10:**
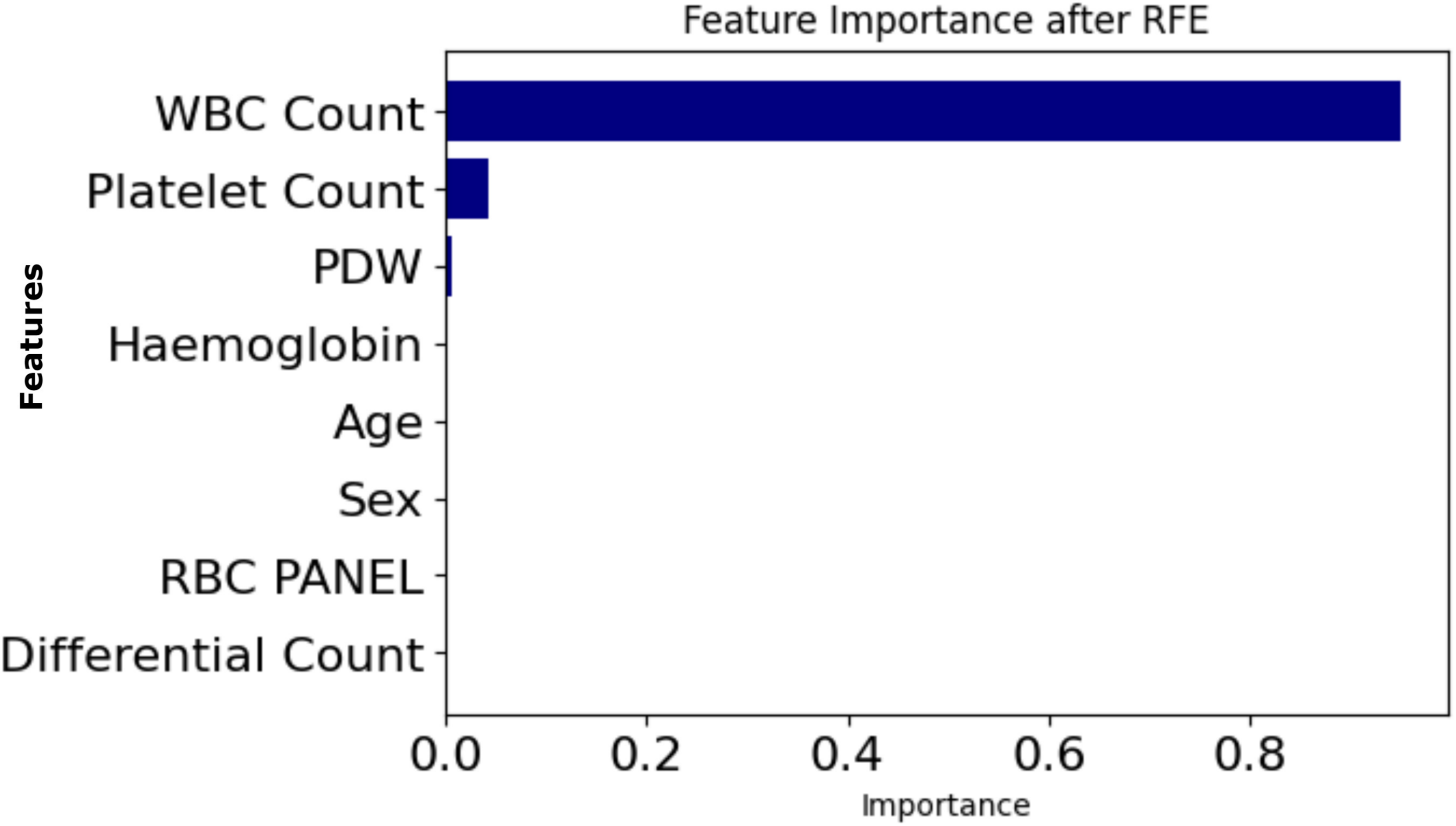
Feature importance based on recursive feature elimination (RFE).

Further supporting this finding is Figure 10, which presents RFE rankings of feature importance. Just like the SHAP output, a similar trend is seen here, where WBC Count ranks highest, followed by Platelet Count and PDW.

SHAP and RFE were applied to the entire dataset, and the similarity of SHAP and RFE results indicates the robustness and stability of the features selected. In addition, the relevant features selected by Genetic Algorithm (GA) based selection are also found to be similar as SHAP and RFE. Robustness, transferability, and ubiquity of these features across different SHAP, RFE, and GA methods align well indicating their dependable clinical indicators by robustness, transferability, and ubiquity.

### 4.12 Multi-perspective explainability strategy

Given the clinical sensitivity of hematological indicators and the potential consequences of misinterpretation, we adopted a structured explainability strategy combining diverse methods from global, local, perturbation-based, and model-driven perspectives. Rather than serving redundant roles, each method offers complementary insight into model behavior for critical features such as *PDW, WBC Count*, and *Platelet Count*.

- **SHAP (Shapley Additive exPlanations)** captured both global and local attributions based on cooperative game theory. It consistently highlighted *WBC Count* and *Platelet Count* as dominant features, and revealed nonlinear dependencies (e.g., between *PDW* and *Hemoglobin*) relevant for joint risk assessment.
- **LIME** offered localized, patient-level interpretations. It proved valuable in low-confidence edge cases, showing how small perturbations in *Platelet Count* could change the predicted outcome when *PDW* values were marginal.
- **Morris sensitivity analysis** quantified input feature influence under systematic noise. It identified *WBC Count* as highly sensitive and robust, while explaining the low gradient-based influence of *PDW*.
- **Permutation importance** measured feature reliance by simulating randomized corruption. It reaffirmed the importance of *WBC Count* and *Platelet Count*, while exposing instability in secondary attributes such as *MCV*.
- **Recursive feature elimination (RFE)** selected a minimal, high-performing subset including *PDW, WBC Count*, and *RDW*, aiding model simplification and informing feature engineering.

This triangulated framework was designed to capture: (i) robustness to perturbation (Morris), (ii) statistical dependency (Permutation), (iii) co-operative and local attribution (SHAP and LIME), and (iv) feature pruning logic (RFE). Discrepancies, such as the differing rankings of *PDW* across methods, were analyzed as informative divergences reflecting methodological contrasts—not inconsistencies. For brevity, SHAP and LIME are emphasized in the main discussion due to their clinical interpretability. Also, Morris sensitivity analysis and permutation importance both assess individual feature impact on model output—Morris by introducing small changes, and permutation by shuffling values and measuring performance drop. In this study, PDW was selected by the Genetic Algorithm (GA) as important, yet both Morris and permutation methods assigned it zero importance. This discrepancy arises because GA evaluates feature combinations, while Morris and permutation assess features individually. Thus, PDW may not be impactful alone but contributes through interactions.

### 4.13 Web application for real time prediction

Finally, after evaluating all the models, it was found that the Decision Tree was the best model for dengue prediction. This model was also used to develop a web application that enables rapid and reliable risk assessment in a user-friendly and accessible way. This application has been designed to help individuals and healthcare professionals make informed decisions about hematological parameters.

**Figure 11:**
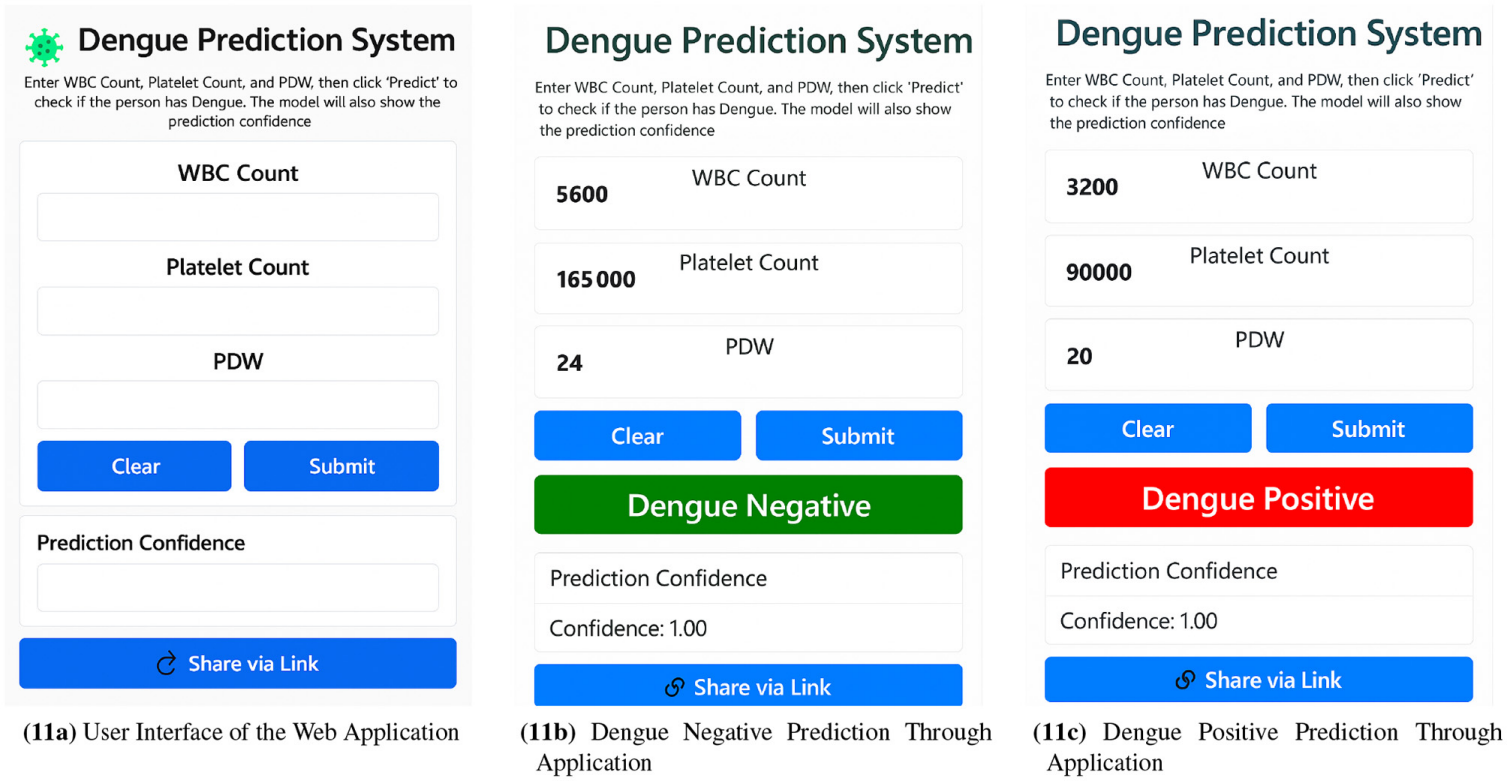
Web application workflow showcasing **(a)** user interface, **(b)** dengue negative prediction, and **(c)** dengue positive prediction via the web application.

The user inference in Figure 11a is self-explanatory. As shown in Figure 11b, the model's prediction of a negative case with 100% confidence and Figure 11c a positive prediction with 100% confidence based on WBC, platelet, and PDW.

Evaluation of several data points shows that, on average, the system takes between 0.4 and 0.6 seconds to make a prediction, which indicates that it can perform in real-time for dengue risk assessment.

### 4.14 Comparative analysis

Compared to other reviewed paper, our study represents a significant advancement in the dengue detection with a novel framework as observed in Table 25. Compared to the conventional methods, our method combines the Explainable AI (XAI), which is essential for making predictions under the trust of and for understanding of by the healthcare professionals. On top of that, our model is deployed as a real-time web application that delivers the insights right away, which most other existing studies don't. One of the significant strengths of our framework is its use of a Genetic Algorithm (GA) to perform feature selection, thereby fine-tuning the identification of the most relevant features that enhance prediction accuracy. We also use Generative Adversarial Networks for data balancing, which helps balance class imbalance and ensures the model's reliability for imbalanced datasets, even those with small datasets. Finally, the Decision Tree model is fine-tuned with hyperparameters at an optimal level to enhance performance without losing transparency. This gives us an impressive 99.49% accuracy with just one misclassification, proving its efficiency. Also, to ensure clean, high-quality data for training and testing, extensive data pre-processing has been performed, which ultimately reduces noise and irrelevant information from the dataset for more reliable predictions. Through this framework, training, testing, and inference operations become faster, which benefits the deployment of real-time clinical applications. Overall, the combination of XAI, real-time deployment, GA-based feature selection, GAN-based data balancing, decision tree optimization, and sound data pre-processing constitutes a novel dengue detection framework with extremely high accuracy, interpretability, and efficiency.

**Table 25:** Summary of existing machine learning studies on dengue detection.

**Study**	**Year**	**Best model**	**Accuracy**	**Data type**	**Limitation**	**XAI**	**Real-time**
Abdualgalil et al. (2022)	2022	Extra tree	99.12%	Tabular	Dataset lacks size/diversity info; no explainability; uses direct test parameters (IgG/IgM).	No	No
Mayrose et al. (2023)	2023	SVM + MobileNetV2	95.74%	Image	Small dataset, limited generalization, no XAI, no real-time support.	No	No
Bohm et al. (2024)	2024	DT, MLP	98%	Tabular	Limited outlier handling, no XAI, unclear features used, no real-time capability.	No	No
Ming et al. (2022)	2022	XGBoost	AUC 0.86	Tabular	Missing data prep details, no explainability methods used.	No	No
Madewell et al. (2025)	2025	CatBoost	AUC 97.1%	Tabular	Incomplete feature description, unclear balancing, no real-time tool.	No	No
Riya et al. (2024)	2023	Stacking ensemble	96.88%	Tabular	Small dataset, high cost, lacks feature interaction, no real-time deployment.	Yes	No
Al-Hagree et al. (2023)	2022	Decision tree	93.7%	Tabular	Small dataset, missing pre-processing/balancing steps, no XAI.	No	Yes
**This study**	**2025**	**Optimized decision tree**	**99.49%**	**Tabular**	**None reported**.	**Yes**	**Yes**

Bold values used to highlight the best-performing results.

### 4.15 System-level justification of model choice: balancing robustness, latency, and interpretability

While numerous deep and ensemble models offer impressive accuracy in medical AI, their practical deployment—especially in low-resource and time-sensitive clinical settings—demands a multidimensional evaluation. We selected Decision Tree (DT) and Artificial Neural Network (ANN) not solely for accuracy but for their superior balance across robustness, latency, and interpretability, as established through statistical evidence, deployment feasibility, and system constraints.

First, statistical robustness was assessed through McNemar's test and paired *t*-tests. The DT model is the only one whose predictions are statistically indistinguishable from ground truth (*p* = 0.6171), while ANN, SVC, and KNN each show significant divergence (*p* < 0.001), as summarized in Table 17 (McNemar's Test Results). Paired *t*-tests against a perfect baseline (Table 18: Paired *t*-test vs. Theoretical Accuracy) indicate no considerable performance gap across all models, with DT (*p* = 0.1108) and ANN (*p* = 0.1039) well above the rejection threshold. This supports our claim that performance parity exists and that more complex models do not guarantee statistically superior generalization under our dataset.

Second, latency—a critical factor in real-world AI deployment—is a key differentiator. As shown in Table 19 (Latency Comparison), DT completes inference in 14.56 ms compared to ANN's 33.52 ms, yielding a 2.3 × reduction. Furthermore, ANN requires more training epochs to converge, thereby increasing resource consumption. This latency advantage is crucial for deployment in time-sensitive applications, such as medical triage or embedded systems, where rapid response directly impacts clinical decision-making.

Third, interpretability remains non-negotiable for responsible clinical AI. DTs offer native rule-based transparency, enabling traceable and auditable decisions by frontline medical staff. In contrast, ANN predictions lack inherent explainability and necessitate *post hoc* interpretive tools such as SHAP or LIME—introducing both computational and cognitive overhead. In field conditions, such overhead reduces usability and impairs accountability. Regulatory frameworks often mandate that algorithmic logic be reviewable by non-technical medical personnel.

Fourth, while more robust architectures—such as CNNs, ensemble forests, or hybrid deep classifiers—may yield marginally better metrics, they are predominantly tailored for image modalities or large-scale signal corpora. Our dataset is tabular and consists of structured clinical measurements. As emphasized by Islam (2025), overparameterized models tend to underperform under distributional shift and lack interpretability. Additionally, studies by Ahamed (2024, 2025) demonstrate the high accuracy of CNN pipelines; however, they require GPU acceleration and involve significant pre-processing, rendering them impractical for deployment in resource-constrained field environments.

In conclusion, the DT model was selected based on consistent statistical robustness, minimum latency, intrinsic interpretability, and practical field deployability. While ANN was retained for its marginally superior AUC, the cumulative evidence supports DT as the optimal model for achieving fast, trustworthy, and explainable clinical decision support under realistic operational constraints.

### 4.16 Clinical impact statement

The given research provides a highly capable, real-time risk prediction tool that meets the demands of practical clinical implementation, particularly triage and screening practices and applications in rural clinical settings. The model can provide interpretable, accurate, and fast assessment based on easily available clinical blood parameters and may considerably improve early detection as well as prioritization of patients in situations of an outbreak of dengue. It can be easily and efficiently calculated and accessed through a web-based interface, which makes it an excellent and feasible tool in a resource-limited environment where diagnostic facilities and highly trained people are rather scarce. In the end, this tool enables frontline healthcare providers and public health authorities to make timely decisions based on data that leads to improved patient outcomes and effective management of outbreaks in different healthcare settings.

## 5 Conclusion and future work

In this study, we design a novel real-time dengue prediction framework based on clinical blood parameters using an optimized Decision Tree. It effectively addresses the limitations of previous machine learning approaches by integrating Genetic Algorithms for feature selection, Generative Adversarial Networks to handle data imbalance, and Explainable AI (XAI) methods to ensure interpretability. Experimental results demonstrate that the proposed Decision Tree model achieves a high classification accuracy of 99.49% with only one misclassification, suggesting strong reliability for clinical decision support. The framework is optimized for fast training, testing, and inference, making it suitable for real-time deployment in healthcare settings. Additionally, a web-based interface was developed to provide rapid and accessible dengue risk assessments, which are particularly valuable during outbreak management scenarios.

Our findings further validate that hematological features such as White Blood Cell (WBC) count and Platelet Count are strong predictors of dengue infection. Although the Genetic Algorithm identified Platelet Distribution Width (PDW) as a relevant feature, subsequent analyses using Morris sensitivity and permutation importance indicated that PDW had a negligible effect on the model's overall predictions. This suggests that PDW may be redundant in this dataset, highlighting the necessity for careful feature evaluation in future studies.

Nonetheless, the model faces significant limitations concerning its generalizability. When tested on a geographically distinct dataset (*n* = 150) with varying IgM/IgG baseline distributions, the F1 score decreased by approximately 12%. This decline underscores the model's sensitivity to domain-specific distributional shifts in hematological markers, a challenge often encountered by clinical AI systems trained on data from homogeneous populations. Future work will incorporate domain adaptation, transfer learning, and uncertainty quantification strategies to enhance robustness and adaptability. Additionally, conducting broader multi-site validations will facilitate a better understanding of inter-institutional variability and assess the framework's effectiveness within diverse public health contexts.

## Data Availability

The original contributions presented in the study are included in the article/supplementary material, further inquiries can be directed to the corresponding authors.
